# Prepregnancy body mass index and risk of preterm birth: association heterogeneity by preterm subgroups

**DOI:** 10.1186/1471-2393-14-153

**Published:** 2014-04-30

**Authors:** Margaret G Parker, Fengxiu Ouyang, Colleen Pearson, Matthew W Gillman, Mandy B Belfort, Xiumei Hong, Guoying Wang, Linda Heffner, Barry Zuckerman, Xiaobin Wang

**Affiliations:** 1 Department of Pediatrics, Boston Medical Center, Boston University School of Medicine, Boston, USA; 2 MOE-Shanghai Key Laboratory of Children’s Environmental Health, Xinhua Hospital, Shanghai Jiaotong University School of Medicine, Shanghai, China; 3 Obesity Prevention Program, Department of Population Medicine, Harvard Medical School/Harvard Pilgrim Health Care Institute, Boston, USA; 4 Department of Nutrition, Harvard School of Public Health, Boston, USA; 5 Division of Newborn Medicine, Department of Pediatrics, Children’s Hospital Boston, Harvard Medical School, Boston, USA; 6 Center on the Early Life Origins of Disease, Department of Population, Family and Reproductive Health, Johns Hopkins University Bloomberg School of Public Health, Baltimore, USA; 7 Department of Pediatrics, Johns Hopkins University School of Medicine, Baltimore, USA; 8 Department of Obstetrics and Gynecology, Boston Medical Center, Boston University School of Medicine, Boston, USA

**Keywords:** Maternal obesity, Prepregnancy BMI, Medically-induced preterm birth, Spontaneous preterm birth, Late preterm birth

## Abstract

**Background:**

To evaluate the association between prepregnancy body mass index (BMI) is associated with early vs. late and medically-induced vs. spontaneous preterm birth (PTB) subtypes.

**Methods:**

Using data from the Boston Birth Cohort, we examined associations of prepregnancy BMI with 189 early (<34 completed weeks) and 277 late (34–36 completed weeks) medically-induced PTBs and 320 early and 610 late spontaneous PTBs vs. 3281 term births (37–44 weeks) in multinomial regression. To assess for mediation by important pregnancy complications, we performed sequential models with and without hypertensive disorders of pregnancy, chorioamnionitis, and gestational diabetes.

**Results:**

Prevalence of prepregnancy obesity (BMI ≥ 30.0 kg/m^2^) was 28% among mothers with medically-induced PTBs, 18% among mothers with spontaneous PTBs, and 18% among mothers with term births (p = <0.001). After adjustment for demographic and known risk factors for PTB, prepregnancy obesity was associated with higher odds of both early [OR 1.78 (1.19, 2.66)] and late [OR 1.49 (1.09, 2.04)] medically-induced PTB. These effect estimates were attenuated with inclusion of hypertensive disorders of pregnancy and gestational diabetes. For spontaneous deliveries, prepregnancy obesity was associated with decreased odds of PTB (0.76 [0.58, 0.98]) and underweight was nearly associated with increased odds of PTB (1.46 [0.99, 2.16]).

**Conclusion:**

Prepregnancy obesity is associated with higher risk of medically-induced, but not spontaneous PTB. Hypertensive disorders of pregnancy and gestational diabetes appear to partially explain the association between prepregnancy obesity and early and late medically-induced PTB.

## Background

In the context of the national obesity epidemic, 1 in 5 pregnant women in the US is obese [1]. Pregnancies among obese women are considered high-risk because they are associated with maternal morbidities including hypertensive and thromboembolic disorders, infection, and gestational diabetes [2]. Concurrent with the rise in obesity prevalence, the rate of preterm birth (PTB) has increased in the past two decades, such that over 12% of infants born in the US are preterm [3]. PTB is the leading cause of neonatal mortality and childhood morbidity, affects over 500,000 infants and costs the US more than $26 billion annually [4,5].

PTB is a heterogeneous condition. PTB can occur spontaneously following preterm labor or premature rupture of membranes or for medical indications. Furthermore, PTB can occur “early” (<34 weeks’ gestation) or “late” (34–36 weeks’ gestation). Pregnancy complications leading to medical inductions disproportionately contribute to late PTB, as compared to early PTB [6-8]. A recent systematic review of 84 studies demonstrated that prepregnancy obesity is associated with a 1.24-fold increased risk of PTB [9]. However, the mechanisms linking prepregnancy obesity with PTB are unclear. Differentiating spontaneous from medically-induced PTB and early from late PTB may offer important clues to understanding the link between prepregnancy obesity and PTB.

Specific complications of pregnancy that occur in obese women may lead to PTB. For example, severe hypertensive disorders of pregnancy are common among obese women, and represent one of the most frequent medical indications for preterm delivery [2]. Spontaneous preterm labor or rupture of membranes is more likely to occur among women with chorioamnionitis [10,11], a condition found more often in obese women compared to normal weight women [12]. Additionally, some evidence suggests that gestational diabetes, another common condition among obese women [2], is associated with medically-induced [13] and spontaneous [13,14] preterm delivery. These findings all suggest potential mechanisms linking prepregnancy obesity with PTB, but few studies have specifically examined the extent to which these conditions mediate observed associations. Additionally, little is known about the relationship of these conditions, and prepregnancy obesity itself, with early versus late PTB, which could also shed light on underlying mechanisms. Our objectives were: (1) to determine the extent to which prepregnancy body mass index (BMI) is associated with early and late medically-induced and spontaneous PTB and (2) to examine the extent to which these relationships are mediated by pregnancy complications related to prepregnancy obesity, specifically hypertensive disorders of pregnancy, chorioamnionitis, and gestational diabetes.

## Methods

### Study population

We studied participants in the Boston Birth Cohort. Briefly, the Boston Birth Cohort is a case–control study designed to evaluate adverse birth outcomes, particularly PTB and intrauterine fetal growth restriction [15]. Beginning in 1998, women have been recruited that are admitted to the Labor and Delivery floor at Boston Medical Center, an inner-city hospital serving predominately minority populations with Medicaid insurance in Boston, Massachusetts. Women have been recruited who delivered a singleton live infant, vaginally or by cesarean section, and meet the definition of a case (gestational age < 37 weeks or birth weight < 2,500 grams) or control (gestational age ≥ 37 completed weeks and birth weight ≥ 2,500 grams). Controls were matched by maternal age and race to cases in a 2:1 ratio. Mother-infant pairs are recruited 24 to 72 hours postpartum. Multiple gestation pregnancies, pregnancies resulting from in-vitro fertilization, deliveries resulting from maternal trauma, and infants born < 23 completed weeks gestation (non-viable) or with major birth defects are excluded. More than 90% of mother-infant pairs approached for study participation enrolled in the study. Recruitment is ongoing. The institutional review board provided ethical approval for this study at Boston Medical Center.

After obtaining written informed consent, mothers are interviewed 24 to 72 hours postpartum using a standardized questionnaire (Additional file 1). A standardized abstraction form is used to obtain clinical data from maternal and infant records, including prenatal care, ultrasound findings, laboratory reports, pregnancy complications, labor and delivery course, and birth outcomes (infant gender, gestational age, and birth weight and length).

**Figure 1:**
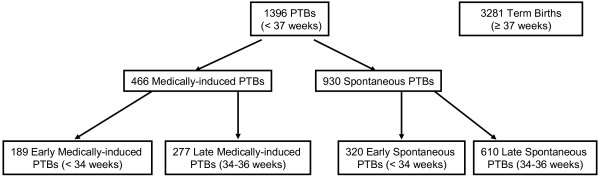
Participant inclusion criteria.

For this analysis, we used mother-infant pairs enrolled from 1998 to 2008. We combined infants born 37–44 completed weeks that are < 2,500 grams (low birth weight, term “cases,” N = 375) and ≥ 2,500 grams (non-low birth weight, term “controls,” N = 2906) to make up our reference group of 3281 term infants. We compared term infants to 1396 preterm infants born at < 37 completed weeks. Of the 1396 preterm cases, we further separated this group into medically-induced (N = 466) and spontaneous (N = 930) PTBs, and according to gestational age at birth (early [<34 completed weeks] and late [34–36 completed weeks] PTBs) in our analyses (Figure 1).

### Main predictor: prepregnancy BMI

Based on maternal self-report of prepregnancy weight and height, we categorized maternal underweight, overweight, and obesity per Institute of Medicine Criteria (IOM) [16] as BMI <18.0 kg/m^2^, 25.0-29.9 kg/m^2^ and BMI ≥ 30.0 kg/m^2^, respectively. Our reference group was normal weight (BMI 18.0-24.9 kg/m^2^).

### Main outcome: PTB

Our main outcome was PTB, categorized by: 1) Delivery type [17], defined as spontaneous (birth to a mother who presented with uterine contractions and/or rupture of membranes) or medically-induced (birth to a mother who presented without uterine contractions or rupture of membranes prior to delivery). Report of maternal presentation prior to delivery was abstracted from the medical record; and 2) Gestational age at the time of delivery, defined as early (<34 completed weeks) or late (34–36 completed weeks) PTB. Gestational age at delivery was determined by maternal report of last menstrual period (LMP) and early fetal ultrasound (<20 weeks). When the ultrasound estimate of gestational age differed by > 7 days than the gestational age predicted by maternal report of LMP, the ultrasound assessment was used. Clinical report of physical exam findings by the attending physician or nurse practitioner was used to estimate gestational age in conjunction with Dubowitz/Ballard Score [18] when no LMP or ultrasound prediction of gestational age was available (N = 12).

### Potential mediators of prepregnancy BMI and PTB

#### Hypertensive disorders of pregnancy

We used clinical blood pressure (BP) measurements and laboratory findings that were abstracted from the maternal medical record before and during pregnancy. We categorized hypertensive disorders of pregnancy according to established guidelines [19,20] as: 1) Chronic hypertension, defined as elevated BP < 20 weeks gestation of the index pregnancy or a clinical diagnosis prior to pregnancy; 2) Mild preeclampsia; 3) Severe hypertensive disorders of pregnancy (severe preeclampsia, eclampsia, and HELLP syndrome; and 4) Normal blood pressure.

#### Chorioamnionitis

We categorized chorioamnionitis based on report of clinical signs and symptoms during labor, as well as placental pathology reports, into the following groups: 1) Clinical chorioamnionitis, defined as maternal temperature > 38°C within 24 hours of delivery in addition to two of the following: uterine tenderness, foul smelling vaginal discharge or amniotic fluid, maternal heart rate >100 beats per minute, fetal heart rate >160 beats per minute, maternal white blood cell count >15,000 cells per cubic liter [21], or placental pathology report of chorioamnionitis; 2) Subclinical chorioamnionitis, defined as any single clinical symptom or sign mentioned above. We created this additional category to test the hypothesis that mild infection may also contribute to PTB; and 3) No chorioamnionitis, defined as none of the symptoms or signs listed above.

#### Gestational diabetes

As a part of clinical care, pregnant women underwent screening for gestational diabetes with an oral non-fasting glucose challenge test and/or fasting glucose tolerance test at 24–28 completed weeks gestation. We formed 3 categories for our analysis: 1) Gestational diabetes; 2) Pre-existing diabetes mellitus (diagnosis prior to 24 weeks gestation of index pregnancy); and 3) No diabetes mellitus. We combined gestational diabetes and diabetes mellitus for multivariate analyses due to small sample sizes in each category.

### Other covariates

Infant sex and birth weight, parity, glucose tolerance, presence of cervical incompetence, and placenta previa were abstracted from the medical record. Report of maternal age, marital status, household income, education level, race/ethnicity, smoking, alcohol and drug use, and stress were obtained during the maternal interview. We calculated gestational weight gain as the difference between the last recorded weight before delivery and the self-reported prepregnancy weight. We categorized women as having gained inadequate, adequate, or excessive weight by week of pregnancy according to the 2009 Institute of Medicine guidelines (0.5-2 kg for the first trimester for all women plus 0.42 kg/week for normal weight women, 0.28 kg/week for overweight women and 0.22 kg/week for obese women in the second and third trimester) [16]. For women who delivered preterm, we determined category of gestational weight gain according to guidelines for the completed week of gestation that delivery occurred.

### Statistical analysis

First we examined bivariate relationships among our main predictor, outcomes, mediators, and other covariates using ANOVA for continuous variables and chi-square tests for categorical variables (Table 1). After testing model assumptions, we first used linear regression to examine the association of prepregancy BMI and gestational weeks at delivery as a continuous variable. Next we used logistic regression to examine odds of PTB vs. term birth according to prepregnancy BMI (Table 2). Then we used multinomial regression to examine odds of medically-induced and spontaneous PTB vs. term birth according to prepregnancy BMI in the same model (Table 2). Finally, also using multinomial regression, we examined odds of early and late medically-induced and early and late spontaneous PTB vs. term birth according to prepregnancy BMI (Table 3). The references group for all models was prepregnancy normal weight.

**Table 1:** Characteristics of Mother-Infant pairs according to delivery type

	**Medically-induced PTBs**^**a**^	**Spontaneous PTBs**^**a**^	**Term births**^**b**^	**p-value**^**c**^
	**(N = 466)**	**(N = 930)**	**(N = 3281)**	
	**Mean (SD)**			
Maternal age (years)	29.5 (6.8)	28.0 (6.6)	27.8 (6.4)	<0.001
Gestational age at birth (weeks)	33.7 (3.0)	33.7 (3.4)	39.5 (1.3)	<0.001
Prepregnancy BMI (kg/m^2^)	27.1 (6.6)	25.5 (6.0)	25.8 (6.1)	<0.001
Birth weight (grams)	1989 (734)	2237 (716)	3239 (563)	<0.001
	**N (%)**			
Prepregnancy BMI (kg/m^2^)				<0.001
<18.5	13 (2.8)	54 (5.8)	149 (4.5)	
18.5-24.9	188 (40.3)	444 (47.7)	1613 (49.2)	
25.0-29.9	136 (29.2)	269 (28.9)	915 (27.9)	
≥ 30.0	129 (27.7)	163 (17.5)	604 (18.4)	
Hypertensive disorders of pregnancy				<0.001
None	179 (38.7)	850 (92.1)	3004 (92.2)	
Chronic hypertension	19 (4.1)	27 (2.9)	71 (2.2)	
Mild preeclampsia	60 (13.0)	24 (2.6)	123 (3.8)	
Severe hypertensive disorders (severe pre-eclampsia, eclampsia, HELLP syndrome)	205 (44.3)	22 (2.4)	61 (1.9)	
Chorioamnionitis				<0.001
None	125 (26.8)	252 (27.1)	1265 (38.6)	
Subclinical chorioamnionitis	117 (25.1)	157 (16.9)	578 (17.6)	
Clinical chorioamnionitis	35 (7.5)	239 (25.7)	302 (9.2)	
Unknown	189 (40.6)	282 (30.3)	1136 (34.6)	
Glucose tolerance				<0.001
No	401 (86.4)	855 (92.3)	3090 (94.5)	
Gestational diabetes	39 (8.4)	46 (5.0)	145 (4.4)	
Diabetes mellitus before index pregnancy	24 (5.2)	25 (2.7)	35 (1.1)	
Gestational weight gain per 2009 IOM				<0.001
Inadequate	50 (10.7)	118 (12.7)	479 (14.6)	
Adequate	38 (8.2)	115 (12.4)	635 (19.4)	
Excessive	148 (31.8)	306 (32.9)	853 (26.0)	
Unknown	230 (49.4)	391 (42.0)	1314 (40.0)	
Married	159 (34.2)	250 (26.9)	1183 (36.2)	<0.001
Household income				0.13
< $30,000/yr	213 (45.7)	475 (51.1)	1580 (48.2)	
≥ $30,000/yr	69 (14.8)	100 (10.8)	387 (11.8)	
Unknown	184 (39.5)	355 (38.2)	1314 (40.0)	
Education				0.02
< High school	138 (29.6)	310 (33.3)	1100 (33.5)	
High school	176 (37.8)	336 (36.1)	1055 (32.2)	
> High school	152 (32.6)	284 (30.5)	1126 (34.3)	
Maternal race/ethnicity				0.003
White	52 (11.2)	130 (14.0)	342 (10.4)	
Black	260 (55.8)	482 (51.8)	1646 (50.2)	
Hispanic	106 (22.7)	213 (22.9)	892 (27.2)	
Other	48 (10.3)	105 (11.3)	401 (12.2)	
Maternal smoking 3 months before or during index pregnancy	98 (21.0)	275 (29.6)	591(18.0)	<0.001
Maternal stress during pregnancy				<0.001
No stress	144 (31.1)	304 (33.1)	1269 (38.9)	
Average stress	194 (41.9)	396 (43.1)	1385 (42.4)	
Very stressful	125 (27.0)	218 (23.7)	612 (18.7)	
Any illicit drug use during pregnancy	76 (16.5)	218 (23.5)	432 (13.2)	<0.001
Alcohol use during pregnancy	30 (6.5)	65 (7.0)	211 (6.4)	0.84
Parity				0.02
Nulliparous	207 (44.4)	385 (41.4)	1381 (42.1)	
Primiparous	119 (25.5)	239 (25.7)	977 (29.8)	
Multiparous	140 (30.0)	306 (32.9)	923 (28.1)	
Cervical incompetence	16 (3.4)	64 (6.9)	37 (1.1)	<0.001
Placenta previa	22 (4.7)	29 (3.1)	32 (1.0)	<0.001
Preterm premature rupture of membranes	0 (0.0)	439 (47.2)	0 (0.0)	N/A
Mode of delivery				<0.001
Vaginal	136 (29.2)	695 (74.8)	2302 (70.2)	
Cesarean section	330 (70.8)	234 (25.2)	977 (29.8)	
Delivery type				N/A
Medically-induced	466 (100.0)	0 (0.0)	971 (29.6)	
Spontaneous	0 (0.0)	930 (100.0)	2310 (70.4)	
PTB according to gestational age			N/A	N/A
Early PTB (<34 weeks)	189 (40.6)	320 (34.4)		
Late PTB (34–36 weeks)	277 (59.4)	610 (65.6)		
Infant sex- female	247 (53.0)	447 (48.1)	1656 (50.5)	0.20

PTB = Preterm Birth; BMI = Body Mass Index; IOM = Institute of Medicine; HELLP = hemolysis, elevated liver enzymes, low platelets.

^a^PTBs include births at 23–36 weeks gestation.

^b^Term Births include births at 37–44 weeks gestation.

^c^P-values calculated from chi-square tests for categorical variables and ANOVA for continuous variables.

**Table 2:** **Odds of medically-induced, spontaneous, and All PTB compared with term birth according to prepregnancy BMI**^**a**^

	**Medically-induced PTB**^**b**^			**Spontaneous PTB**^**b**^			**All PTB**^**c**^		
***Crude and adjusted models***	OR	95% C.I.	p-value	OR	95% C.I.	p-value	OR	95% C.I.	p-value
M1: Unadjusted									
< 18.5	0.75	0.42, 1.35	0.33	1.32	0.95, 1.83	0.10	1.15	0.85, 1.55	0.37
18.5-24.9 (ref)	1.00			1.00			1.00		
25.0-29.9	1.28	1.01, 1.61	0.04	1.07	0.90, 1.27	0.45	1.13	0.97, 1.31	0.11
≥ 30.0	1.83	1.44, 2.34	<0.001	0.98	0.80, 1.20	0.85	1.23	1.04, 1.46	0.01
M2: Adjusted^d^									
<18.5	0.94	0.52, 1.71	0.84	1.40	0.99, 1.99	0.06	1.29	0.94, 1.77	0.12
18.5-24.9 (ref)	1.00			1.00			1.00		
25.0-29.9	1.11	0.87, 1.43	0.39	0.99	0.82, 1.18	0.87	1.02	0.87, 1.20	0.79
≥ 30.0	1.59	1.23, 2.05	<0.001	0.90	0.73, 1.12	0.35	1.11	0.93, 1.33	0.25
***Models to further assess mediation***									
M3: M2 + hypertensive disorders of pregnancy^e^									
<18.5	1.19	0.60, 2.34	0.62	1.41	1.00, 2.00	0.05	1.38	0.99, 1.91	0.05
18.5-24.9 (ref)	1.00			1.00			1.00		
25.0-29.9	0.96	0.72, 1.29	0.81	0.98	0.81, 1.17	0.80	0.97	0.83, 1.15	0.76
≥ 30.0	1.13	0.82, 1.54	0.46	0.91	0.73, 1.13	0.40	0.97	0.80, 1.17	0.73
M4: M2 + chorioamnionitis^f^									
<18.5	0.93	0.51, 1.69	0.81	1.48	1.04, 2.10	0.03	1.32	0.96, 1.82	0.09
18.5-24.9 (ref)	1.00			1.00			1.00		
25.0-29.9	1.12	0.87, 1.43	0.39	0.99	0.83, 1.20	0.95	1.03	0.88, 1.21	0.73
≥ 30.0	1.58	1.22, 2.05	0.001	0.91	0.73, 1.12	0.37	1.12	0.93, 1.34	0.23
M5: M2 + gestational diabetes^f^									
<18.5	0.96	0.53, 1.75	0.90	1.42	1.00, 2.01	0.05	1.31	0.95, 1.80	0.10
18.5-24.9 (ref)	1.00			1.00			1.00		
25.0-29.9	1.06	0.82, 1.36	0.66	0.96	0.79, 1.15	0.63	0.98	0.84, 1.15	0.83
≥ 30.0	1.40	1.08, 1.83	0.01	0.86	0.69, 1.07	0.16	1.03	0.85, 1.23	0.78
***Grand model***									
M6: M2 + hypertensive disorders of pregnancy^e^ + chorioamnionitis^f^ + gestational diabetes^g^									
<18.5	1.21	0.61, 2.39	0.58	1.51	1.06, 2.15	0.02	1.45	1.04, 2.02	0.03
18.5-24.9 (ref)	1.00			1.00			1.00		
25.0-29.9	0.94	0.70, 1.27	0.69	0.95	0.79, 1.15	0.60	0.95	0.80, 1.12	0.54
≥ 30.0	1.08	0.78, 1.49	0.65	0.86	0.69, 1.07	0.17	0.92	0.75, 1.11	0.38

BMI = Body Mass Index; PTB = Preterm Birth.

^a^Data from 466 mothers with medically-induced PTBs, 930 mothers with spontaneous PTBs, and 3281 mothers with term births.

^b^Multinomial regression used to compare medically-induced and spontaneous PTB with term birth (reference group).

^c^Logistic regression used to compare all PTB with term birth (reference group).

^d^Adjusted model includes maternal age, race/ethnicity, marital status, income, education, child sex, maternal gestational weight gain, smoking, alcohol or illicit drug use, stress, parity, cervical incompetence, and placenta previa.

^e^Hypertensive disorders of pregnancy are categorized as chronic hypertension, mild preeclampsia, severe hypertensive disorders (severe preeclampsia, eclampsia, HELLP syndrome), and none.

^f^Chorioamnionitis is categorized as subclinical chorioamnionitis, clinical chorioamnionitis, and none.

^g^Maternal diabetes is categorized as gestational diabetes or diabetes prior to pregnancy vs. none.

**Table 3:** **Odds of early and late medically-induced and spontaneous PTB compared with term birth according to prepregnancy BMI**^**a,b**^

	**Early medically-induced PTB**			**Late medically-induced PTB**			**Early spontaneous PTB**			**Late spontaneous PTB**		
***Crude and adjusted models***	OR	95% C.I.	p-value	OR	95% C.I.	p-value	OR	95% C.I.	p-value	OR	95% C.I.	p-value
M1: Unadjusted												
< 18.5	0.33	0.08, 1.37	0.13	0.97	0.51, 1.83	0.92	1.30	0.76, 2.20	0.34	1.33	0.91, 1.94	0.15
18.5-24.9 (ref)	1.00			1.00			1.00			1.00		
25.0-29.9	1.95	1.38, 2.76	<0.001	0.92	0.67, 1.25	0.59	1.12	0.85, 1.47	0.43	1.04	0.85, 1.28	0.67
≥ 30.0	2.05	1.40, 3.01	<0.001	1.72	1.27, 2.31	<0.001	1.34	0.99, 1.80	0.06	0.81	0.63, 1.05	0.11
M2: Adjusted^c^												
<18.5	0.43	0.10, 1.80	0.25	1.18	0.61, 2.27	0.62	1.30	0.73, 2.30	0.37	1.46	0.99, 2.16	0.06
18.5-24.9 (ref)	1.00			1.00			1.00			1.00		
25.0-29.9	1.68	1.16, 2.41	0.01	0.81	0.59, 1.13	0.21	1.02	0.76, 1.37	0.90	0.98	0.79, 1.21	0.84
≥ 30.0	1.78	1.19, 2.66	0.01	1.49	1.09, 2.04	0.01	1.25	0.90, 1.72	0.18	0.76	0.58, 0.98	0.04
***Models to assess mediation***												
M3: M2 + hypertensive disorders of pregnancy^d^												
<18.5	0.63	0.14, 2.84	0.55	1.35	0.66, 2.76	0.41	1.31	0.74, 2.33	0.35	1.47	0.99, 2.17	0.06
18.5-24.9 (ref)	1.00			1.00			1.00			1.00		
25.0-29.9	1.52	1.00, 2.33	0.05	0.73	0.52, 1.04	0.08	1.02	0.75, 1.37	0.91	0.97	0.78, 1.19	0.74
≥ 30.0	1.17	0.73, 1.87	0.53	1.12	0.79, 1.59	0.53	1.27	0.92, 1.75	0.15	0.76	0.58, 0.99	0.04
M4: M2 + chorioamnionitis^e^												
<18.5	0.42	0.10, 1.77	0.24	1.17	0.61, 2.26	0.63	1.48	0.81, 2.70	0.20	1.48	1.00, 2.19	0.05
18.5-24.9 (ref)	1.00			1.00			1.00			1.00		
25.0-29.9	1.70	1.18, 2.45	0.01	0.81	0.59, 1.12	0.21	1.04	0.76, 1.42	0.82	0.98	0.79, 1.21	0.86
≥ 30.0	1.77	1.18, 2.64	0.01	1.49	1.09, 2.04	0.01	1.30	0.93, 1.83	0.13	0.76	0.58, 0.98	0.04
M5: M2 + gestational diabetes^f^												
<18.5	0.44	0.10, 1.82	0.26	1.22	0.63, 2.33	0.56	1.32	0.74, 2.34	0.34	1.48	1.00, 2.19	0.05
18.5-24.9 (ref)	1.00			1.00			1.00			1.00		
25.0-29.9	1.64	1.14, 2.36	0.01	0.75	0.54, 1.04	0.09	0.99	0.73, 1.33	0.93	0.95	0.77, 1.18	0.64
≥ 30.0	1.68	1.12, 2.52	0.01	1.25	0.90, 1.74	0.18	1.16	0.84, 1.61	0.37	0.73	0.56, 0.95	0.02
***Grand model***												
M6: M2 + hypertensive disorders of pregnancy^d^ + chorioamnionitis^e^ + gestational diabetes^f^												
<18.5	0.63	0.14, 2.86	0.55	1.39	0.68, 2.82	0.36	1.52	0.83, 2.78	0.17	1.50	1.01, 2.22	0.04
18.5-24.9 (ref)	1.00			1.00			1.00			1.00		
25.0-29.9	1.58	1.03, 2.42	0.04	0.69	0.49, 0.99	0.04	0.99	0.72, 1.37	0.98	0.94	0.76, 1.16	0.56
≥ 30.0	1.22	0.76, 1.98	0.41	1.01	0.70, 1.45	0.96	1.19	0.84, 1.69	0.33	0.73	0.56, 0.95	0.02

BMI = Body Mass Index; PTB = Preterm Birth.

^a^Data from 189 mothers with early medically-induced PTBs, 277 mothers with late medically-induced PTBs, 320 mothers with early spontaneous PTBs, 610 mothers with late spontaneous PTBs, and 3281 mothers with term births.

^b^Multinomial regression used to compare early and late medically-induced and spontaneous PTB with term birth (reference group).

^c^Adjusted model includes maternal age, race/ethnicity, marital status, income, education, child sex, maternal gestational weight gain, smoking, alcohol or illicit drug use, stress, parity, cervical incompetence, and placenta previa.

^d^Hypertensive disorders of pregnancy are categorized as chronic hypertension, mild preeclampsia, severe hypertensive disorders (severe preeclampsia, eclampsia, HELLP syndrome), and none.

^e^Chorioamnionitis is categorized as subclinical chorioamnionitis, clinical chorioamnionitis, and none.

^f^Maternal diabetes is categorized as gestational diabetes or diabetes prior to pregnancy vs. none.

Model 1 included prepregnancy BMI only. Model 2 additionally included demographic variables (maternal age, race/ethnicity, marital status, income, education, child sex) and variables that are associated with PTB [10] (maternal smoking, alcohol and drug use, stress, cervical incompetence, placental previa, and gestational weight gain). In Model 3–5, we sequentially added hypertensive disorders of pregnancy, chorioamnionitis, and gestational diabetes to examine potential mediating effects of these variables on the relationship between prepregnancy BMI and PTB. In Model 5, we included all covariates and mediators to assess if the combination of these factors changes the main associations. We performed data analysis with SAS 9.3 (SAS Institute, Cary, North Carolina).

## Results

Participant characteristics are shown in Table 1. Among 1396 PTBs, 466 (33%) were medically-induced PTBs and 930 (67%) were spontaneous PTBs. Prevalence of prepregnancy obesity was 28% among mothers with medically-induced PTBs, 18% among mothers with spontaneous PTBs, and 18% among mothers with term births (Table 1). Severe hypertensive disorders of pregnancy and gestational diabetes were most prevalent among mothers with medically-induced PTBs (44% and 8%), compared to mothers with spontaneous PTBs (2% and 5%), and term births (2% and 4%). Clinical chorioamnionitis was most prevalent among mothers of spontaneous PTBs (30%), compared to mothers with medically-induced PTBs (8%), and term births (9%) (Table 1).

After adjusting for demographic variables and confounders, we found that, among all subjects, mothers with prepregnancy obesity delivered infants 0.28 weeks (95% C.I. [-0.54 to -0.03]) earlier than mothers with prepregnancy normal weight (data not shown). We found that this relationship was attenuated when hypertensive disorders of pregnancy (-0.28 to -0.07) and diabetes (-0.28 to -0.17), but not when chorioamnionitis (-0.28 to -0.30) was added to our model (data not shown). We found no associations between prepregnancy BMI and all subtypes of PTB (OR 1.11 [0.93, 1.33]) (Table 2, Model 2). When we examined medically-induced and spontaneous PTBs separately, we found that prepregnancy obesity was associated with a 1.6-fold (95% C.I. 1.23, 2.05) increased odds of medically-induced PTB, but not spontaneous PTB (OR 0.90 [95% C.I. 0.73, 1.12]), compared to term birth (Table 2, Model 2). The association between prepregnancy obesity and medically-induced PTB was largely attenuated after adding hypertensive disorders of pregnancy (OR 1.59 to 1.13) and gestational diabetes (OR 1.59 to 1.40) to our model (Table 2, Model 3 and 5), and in our “grand” model (OR 1.59 to 1.08) (Table 2, Model 6), which included all mediators and covariates. We also found an increased odds of spontaneous PTB among underweight mothers in our adjusted model (1.40 [95% C.I. 0.99, 1.99]) (Table 2, Model 2) and in our grand model (1.51 [95% C.I. 1.06, 2.15]) (Table 2, Model 6).

Finally, we examined early and late medically-induced and early and late spontaneous PTB vs. term birth (Table 3). Among medically-induced PTBs, we found that prepregnancy obesity was associated with increased odds of early (1.78 [1.19, 2.66]) and late 1.49 [1.09, 2.04]) PTB (Table 3, Model 2). The relationships between prepregnancy obesity and early and late medically-induced PTB were attenuated with inclusion of hypertensive disorders of pregnancy (OR 1.78 to 1.17 for early and OR 1.49 to 1.12 for late PTB)(Table 3, Model 2 and 3) and gestational diabetes (OR 1.78 to 1.68 for early and OR 1.49 to 1.25 for late PTB) (Table 3, Model 2 and 5). Among spontaneous PTBs, we found no associations between prepregnancy BMI and early spontaneous PTB, but we found associations between maternal underweight and obesity and late spontaneous PTB in opposite directions. Maternal underweight was associated with an increased odds (1.46 [0.99, 2.16]) of late spontaneous PTB and maternal obesity was associated with a decreased odds (0.76 [0.58, 0.98]) of late spontaneous PTB (Table 3, Model 2). We additionally show the direction of the associations of prepregnancy underweight and obesity with early and late medically-induced and spontaneous PTB with arrows in Table 4.

**Table 4:** Direction of associations between maternal underweight and obesity and early and late medically-induced and spontaneous PTB compared to term births

	**Medically-induced PTB**		**Spontaneous PTB**	
	**Early**	**Late**	**Early**	**Late**
Maternal underweight	─	─	─	↑
Maternal obesity	↑	↑	─	↓

─no association; ↑ = increased odds; ↓ = decreased odds.

Finally, we performed a sensitivity analysis excluding term, low birth weight (≤2,500 g) infants in our reference group, as these infants were “cases” in the original case–control study design of the Boston Birth Cohort. We found similar results examining prepregnancy BMI with 2906 term, non-low birth weight (>2,500 g) “controls,” so we show results without combined reference group of 3281 term infants.

## Discussion

In this US inner-city study of mother-infant pairs, we found that prepregnancy obesity was associated with a higher risk of medically-induced PTB, both early (<34 weeks’ gestation, 1.8-fold higher risk) and late (34–36 weeks’ gestation, 1.5-fold higher risk). These associations were explained in part by hypertensive disorders of pregnancy and less so by gestational diabetes, common co-morbidities of obesity in pregnancy that are also associated with medically-induced PTB. In contrast, prepregnancy obesity was not associated with early spontaneous PTB, and inversely associated with late spontaneous PTB. We also found that prepregnancy underweight was associated with higher risk of spontaneous late PTB.

While many studies have reported that maternal obesity is a risk factor for PTB [9], fewer have separated medically-induced from spontaneous PTB. Our findings agree with other studies linking prepregnancy obesity with an increased risk of medically-induced PTB [22-28]. We extended findings of prior studies by examining specific reasons for medical induction. In our study, hypertensive disorders of pregnancy and gestational diabetes largely explained the association of prepregnancy obesity with both early and late medically induced PTB, suggesting that these conditions may lie on the causal pathway between maternal obesity and PTB. These results suggest that interventions targeted at reducing severity of hypertensive disorders of pregnancy and gestational diabetes may reduce risks of medically-induced PTB among obese women.

In contrast to the more consistent evidence linking prepregnancy obesity with medically-induced PTB, studies of spontaneous PTB have yielded inconsistent results. Among studies examining associations of prepregnancy obesity and *early* spontaneous PTB, three reported higher risk [24,26,27], one reported lower risk [25], whereas we found no association. Among studies examining associations of prepregnancy obesity and *late* spontaneous PTB, two reported higher risk [27,29], one reported no association [24], whereas we found in our cohort a surprising *lower* risk. Lack of consistency in findings could be due to lack of adjustment by factors associated with PTB such as gestational weight gain [25,26], smoking [25], or illicit drug use [25], or due to inconsistent cut points of maternal BMI to define obesity and gestational age to define “early” and “late” PTB. For instance, we defined prepregnancy obesity per Institute of Medicine Criteria [16] as BMI ≥ 30.0 kg/m^2^, while others examined more granular BMI subgroups (i.e. BMI 30.0-34.9 kg/m^2^, 35.0-39.9 kg/m^2^, and ≥ 40.0 kg/m^2^) [24,26]. It is possible that our null association of prepregnancy obesity (a composite of all mothers with a prepregnancy BMI ≥ 30.0 kg/m^2^) and early spontaneous PTB, may not adequately capture associations that may be present among extremely obese women only. Furthermore, we defined late preterm as infants born 34–36 weeks’ gestation according to the American Academy of Pediatrics [30,31] and American College of Obstetrics and Gynecology [31], and combined all infants born < 34 weeks gestation as “early” PTB. Others defined later PTB subgroups as 35-37 [24] or 32-36 [29] weeks’ gestation. Inconsistent findings among studies that use different gestational age cut points suggest that different mechanisms of spontaneous PTB may occur at various stages of gestation. Finally, it is possible that associations of maternal obesity and spontaneous PTB are inconsistent because of variable clinical thresholds for medical-induction. Obese women who are at risk for spontaneous PTB may instead deliver following medical-induction because of clinical concerns that differ by study population, thus changing the remaining pool of women at risk for spontaneous PTB.

Some authors [26,27] have hypothesized that chorioamnionitis may be on the causal pathway between prepregnancy obesity and PTB. We specifically addressed this hypothesis by examining chorioamnionitis as a mediating factor between prepregnancy obesity and spontaneous PTB. However, we did not find higher risks of spontaneous PTB among obese women, precluding the ability to appreciate a mediating effect by chorioamnionitis. Future studies that evaluate inflammatory markers, other infectious conditions besides chorioamnionitis, or additional factors associated with prepregnancy obesity, are needed to further uncover mechanisms spontaneous PTB among obese women. Because mechanisms of spontaneous PTB may vary throughout pregnancy, a continuum of gestational ages should be examined.

On the other end of the weight spectrum, numerous studies have reported an increased risk of spontaneous PTB among underweight women [13,22,24,25,27]. In our study, maternal underweight was associated with a 1.5-fold higher odds of *late* (but not *early*) spontaneous PTB, independent of gestational weight gain. We speculate that poor maternal nutritional status in pregnancy is particularly costly to the fetus during late pregnancy, a period of rapid fetal growth and nutrient accretion. In contrast, maternal underweight was not associated with an increased risk for medically indicated PTB.

Strengths of this study include careful adjustment for many known risk factors of PTB, including demographic variables and maternal pregnancy conditions, including gestational weight gain. Our study population was composed of US, inner-city, predominantly low soceioeconomic and minority mother-infant pairs enabling us to examine associations of prepregnancy BMI and several subtypes of PTB, as well as potential mediating factors, among mothers with high risk of PTB. Our population may not be generalizeable to others. Other limitations include maternal self-report of weight and height to calculate prepregnancy BMI. Under reporting of weight or over reporting height may have lead to falsely decreased BMI, as described in other studies [32], and may have biased our analyses examining prepregnancy overweight and obesity toward the null, and prepregnancy underweight away from the null.

## Conclusion

We found that maternal obesity is associated with higher risk of medically-induced but not spontaneous PTB, and further, that hypertensive disorders of pregnancy and gestational diabetes appear to largely explain this association. Our findings suggest that intervention strategies targeted at reducing severity or incidence of severe hypertensive disorders and gestational diabetes among obese women may, in turn, reduce medically-induced PTB. In contrast, the relationship between maternal obesity and spontaneous PTB remains unclear. Future studies that examine factors along the causal pathway between maternal obesity and early and late spontaneous PTB subgroups are needed to identify targets for intervention.

## Abbreviations

BP: Blood pressure; BMI: Body mass index; HELLP: Hemolysis, elevated liver enzymes, low platelets; IOM: Institute of Medicine; LMP: Last menstrual period; PTB: Preterm birth; PPROM: Preterm premature rupture of membranes.

## Competing interests

The authors declare that they have no competing interests.

## Authors’ contributions

MP (corresponding author) was responsible for conception and design of this secondary analysis of a longitudinal cohort study, contributed to the statistical analysis, and was heavily involved in drafting and editing all versions of the manuscript. FO contributed to study design and performed all statistical analysis. CP coordinated all data collection and participant recruitment. XH and GW contributed to data collection and statistical analysis. MG, MB, BZ, FH, and XW contributed to the conception of the study. MG, MB, BZ, and XW contributed to the drafting and editing of the manuscript. All authors read and approved the final manuscript.

## Pre-publication history

The pre-publication history for this paper can be accessed here:

http://www.biomedcentral.com/1471-2393/14/153/prepub

## Supplementary Material

Additional file 1 Maternal Postpartum Questionnaire. Click here for file
