# Supplementary material for: Prepregnancy body mass index and risk of preterm birth: association heterogeneity by preterm subgroups
Source: BMC Pregnancy Childbirth. 2014 Apr 30;14:153. doi: 10.1186/1471-2393-14-153 (PMC4022544; doi:10.1186/1471-2393-14-153)
Supplement: Additional file 1 — Maternal Postpartum Questionnaire. [file 1471-2393-14-153-S1.pdf]

8308467118

Boston Medical Center

MATERNAL POSTPARTUM QUESTIONNAIRE

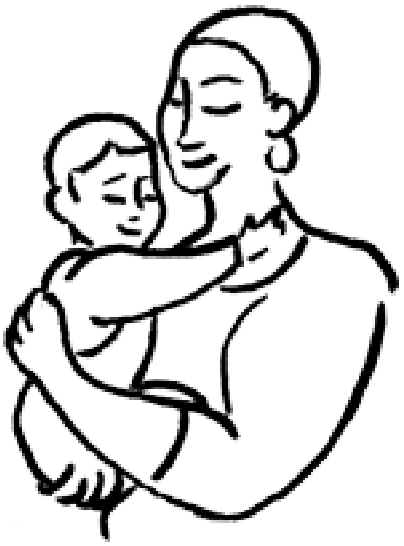

Study ID ____________

Today's date __ __ / __ __ / __ __

Interview is   ○ Complete   ○ Incomplete

Location of Interview ____________

Interviewer's Name ____________

If incomplete date of future visit __ __ / __ __ / __ __

Revised on 04/29/09

8483467112

Study ID______________________________

Protocol #98 - 38

" Molecular Epidemiologic Study of Low Birth Weight "

I attest that I have fully and appropriately informed this subject of the nature of the above research study and have offered to answer any question that she may have. This subject has agreed to participate in the study and signed the written informed consent form.

______________________________________ ____________________

Signature of Principal Investigator/Designate Date

Study ID___________________________

1. Interview conducted:

○ Alone ○ Friends in room ○ Father of baby in room ○ Interpreter in room

Interviewers: please read the following statement to the subject before you begin interview. I WOULD LIKE TO REMIND YOU THAT YOU MAY SKIP ANY QUESTION YOU DO NOT WISH TO ANSWER. The following questions are about your general health before and during this pregnancy.

I. GENERAL HEALTH STATUS

2. Your prepregnancy height [ ] **feet** [ ][ ] **inches (or** [ ][ ][ ] **cm)**

3. Your prepregnant weight [ ][ ][ ] **lbs (or** [ ][ ][ ] **kg)**

4. Your total weight gain during pregnancy [ ][ ][ ] **lbs (or** [ ][ ][ ] **kg)**

5. Can I ask you about your child's biological father's height, weight, and age? ○ Yes ○ No

If NO, skip to question 6 below

5a. Baby's father's height [ ] **feet** [ ][ ] **inches (or** [ ][ ][ ] **cm)**

5b. Baby's father's weight [ ][ ][ ] **lbs (or** [ ][ ][ ] **kg)**

5c. Baby's father's age [ ][ ] **years**

II. INFORMATION ABOUT THIS INDEX PREGNANCY

6. Did you have a vaginal or C-Section delivery? ○ Vaginal ○ C/S

When you came to the hospital, was your first sign of labor

○ Uterine CTX ○ ROM without CTX ○ Both CTX and ROM ○ None of the above

If " None of the above",

Was you labor induced by your doctor/midwife during this pregnancy? ○ Yes ○ No

7. Did you get any prenatal care from a doctor or nurse-midwife during this pregnancy?

○ Yes ○ No

If yes, then go to next questions, if NO, SKIP TO QUESTION 13

8. Where did you get your prenatal care?

○ BMC--Women's Center ○ BMC--Doctor's Office Building ○ BMC--Adolescent Center ○ Other

If "Other", specify: [ ]

9. When did you find out you were pregnant? [ ][ ] **wks gestation**

10. How many weeks pregnant were you when you went for your first prenatal visit? [ ][ ] **wks**

11. How many prenatal appointments did you miss? ☐☐ **appointments**

11a. How many prenatal appointments did you have?
O less than 5 O 5 - 10 O more than 10 visits

11b. How many prenatal ultrasounds did you have? ☐☐

12. Did you have any flu during this pregnancy? O Yes O No

a. First trimester O Yes O No

b. Second trimester O Yes O No

c. Third trimester O Yes O No

13. Did you have any fever during this pregnancy? O Yes O No

a. First trimester O Yes O No

b. Second trimester O Yes O No

c. Third trimester O Yes O No

14. During this pregnancy, did you have any swelling, water retention, or edema? O Yes O No

a. Did your ankles swell? O Yes O No **If yes, starting at** ☐☐ **weeks**

b. Did your legs swell? O Yes O No **If yes, starting at** ☐☐ **weeks**

c. Did your hands swell? O Yes O No **If yes, starting at** ☐☐ **weeks**

d. Did your face swell? O Yes O No **If yes, starting at** ☐☐ **weeks**

15. Do you or have you ever had any history of asthma?
O No O Only when I was a child, but outgrew now O Yes, I have it now

If yes, did you experience any asthma attacks during your pregnancy? O Yes O No

a. 1st trimester? O Yes O No **If yes, how many times** ☐☐

b. 2nd trimester O Yes O No **if yes, how many times** ☐☐

c. 3rd trimester O Yes O No **if yes, how many times** ☐☐

16. Do you or have you ever had Eczema?
O No O Only when I was a child, but outgrew now O Yes, I have it now

17. Do you or have you ever had hay fever or seasonal allergies?
O No O Only when I was a child, but outgrew now O Yes, I have it now

18. Do you have any drug allergies? O Yes O No **(please use "," to separate drugs)**

If yes, specify the drug: [ ]

19. Do you or have you ever had food or environmental allergy? (If no, skip to section III)

O No O Only when I was a child, but outgrew now O Yes, I have it now

If you ever had an allergy, what type of allergen are you allergic to?

19a. Cow's milk (including cheese and diary products) O Yes O No

19b. Egg O Yes O No

19c. Peanut O Yes O No

19d. Walnut O Yes O No

19e. Sesame O Yes O No

19f. Shellfish O Yes O No

19g. Fish O Yes O No

19o. Others O Yes O No

19h. Soy O Yes O No

19i. Wheat O Yes O No

19j. Cat O Yes O No

19k. Dog O Yes O No

19l. Cockroach O Yes O No

19m. Dust Mites O Yes O No

19n. Molds O Yes O No

If Others, specify: [ ] [ ] [ ] [ ] [ ] [ ]

III. Allergy related conditions in baby's father

20. Can I ask you some allergy related questions about your baby's father? O Yes O No

If NO, skip to question 26 (next page)

21. Does he or has he ever had Eczema?

O No O Only when he was a child, but outgrew now O Yes, he has it now O Don't know

22. Does he or has he ever had any history of asthma?

O No O Only when he was a child, but outgrew now O Yes, he has it now O Don't know

23. Does he or has he ever had hay fever or seasonal allergies?

O No O Only when he was a child, but outgrew now O Yes, he has it now O Don't know

24. Does he or has he ever had any drug allergies? ○ Yes ○ No ○ Don't know

If yes, specify the drug: [ ]

(please use "," to separate drugs)

25. Does he or has he ever had any food or environmental allergies?
○ No ○ Only when he was a child, but outgrew now ○ Yes, he has it now ○ Don't know

If NO, skip to question 26 below

If he ever had a allergy, what type of allergen is he allergic to?

25a. Cow's milk (including cheese and diary products) ○ Yes ○ No

25b. Egg ○ Yes ○ No

25c. Peanut ○ Yes ○ No

25d. Walnut ○ Yes ○ No

25e. Sesame ○ Yes ○ No

25f. Shellfish ○ Yes ○ No

25g. Fish ○ Yes ○ No

25o. Others ○ Yes ○ No

25h. Soy ○ Yes ○ No

25i. Wheat ○ Yes ○ No

25j. Cat ○ Yes ○ No

25k. Dog ○ Yes ○ No

25l. Cockroach ○ Yes ○ No

25m. Dust Mites ○ Yes ○ No

25n. Molds ○ Yes ○ No

If Others, specify: [ ] [ ] [ ] [ ] [ ] [ ]

26. During this pregnancy, did you have any vaginal bleeding? ○ Yes ○ No

a. During first trimester ○ Yes ○ No

b. During second trimester ○ Yes ○ No

c. During third trimester ○ Yes ○ No

d. Preceding labor and delivery ○ Yes ○ No

9408467110 **Study ID**______________________________

27. Did you have any vaginal or genital tract or urinary tract infections during pregnancy? (including yeast infections)

O Yes O No

If YES: I'd like to ask you some questions about those infections. How many times did you have an infection? What kinds of infections were they?

**Infection Details**	**1st Time**	**2nd Time**	**3rd Time**	**4th Time**
**a. Trimester**	O 1 O 2 O 3	O 1 O 2 O 3	O 1 O 2 O 3	O 1 O 2 O 3
**b. Type**	O Chlamydia<br>O Gonorrhea<br>O Syphilis<br>O Trichomonas<br>O GBS<br>O BV<br>O Yeast<br>O Herpes<br>O HPV<br>O Other GT<br>O Unknown GTI<br>O Urinary Tract	O Chlamydia<br>O Gonorrhea<br>O Syphilis<br>O Trichomonas<br>O GBS<br>O BV<br>O Yeast<br>O Herpes<br>O HPV<br>O Other GT<br>O Unknown GTI<br>O Urinary Tract	O Chlamydia<br>O Gonorrhea<br>O Syphilis<br>O Trichomonas<br>O GBS<br>O BV<br>O Yeast<br>O Herpes<br>O HPV<br>O Other GT<br>O Unknown GTI<br>O Urinary Tract	O Chlamydia<br>O Gonorrhea<br>O Syphilis<br>O Trichomonas<br>O GBS<br>O BV<br>O Yeast<br>O Herpes<br>O HPV<br>O Other GT<br>O Unknown GTI<br>O Urinary Tract
**c. Specify Other GT**				
**d. What was the treatment you received?**	O None<br>O Pill<br>O Shot<br>O Cream<br>O Other	O None<br>O Pill<br>O Shot<br>O Cream<br>O Other	O None<br>O Pill<br>O Shot<br>O Cream<br>O Other	O None<br>O Pill<br>O Shot<br>O Cream<br>O Other
**e. How much of the treatment did you take?**	O None<br>O Some<br>O All	O None<br>O Some<br>O All	O None<br>O Some<br>O All	O None<br>O Some<br>O All
**f. Specify Other treatment**				

28. Thinking back, just before you became pregnant, did you want to become pregnant at that time?

O Yes O No

IF NO: 28a. Do you want to become pregnant in the future? O Yes O No

29. How would you characterize the amount of stress in your life in general?

O Not stressful O Average O Very Stressful

30. How would you characterize the amount of stress in your life during this pregnancy?

O Not stressful O Average O Very Stressful

31. In the last month, how often have you felt that you were unable to control the important things in your life?

O Never O Almost never O Sometimes O Fairly often O Very often

32. In the last month, how often have you felt confident about your ability to handle your personal problems?

O Never O Almost never O Sometimes O Fairly often O Very often

33. In the last month, how often have you felt that things were going your way?

O Never O Almost never O Sometimes O Fairly often O Very often

34. In the last month, how often have you felt difficulties were piling up so high that you could not overcome them?

O Never O Almost never O Sometimes O Fairly often O Very often

35. Did you experience any major stressful events, such as lost family members, divorce, lost job, severe illness/injuries of family member, etc? Mark for each time period. O Yes O No

a. Prepregnancy(within 1 year of conception) O Yes O No **Specify:**

b. 1st Trimester O Yes O No **Specify:**

c. 2nd Trimester O Yes O No **Specify:**

d. 3rd Trimester O Yes O No **Specify:**

36. Did you witness any violence during your pregnancy? O Yes O No

IF YES, Specify:

36a. If YES, was it: O Inside your home O Outside your home O Both

37. How would you describe the amount of involvement there was during your pregnancy from the father of your baby? Would you say he was:

O Not involved O A little involved O Mostly involved O Very involved

38. How would you rate the amount of social support you received from the father of your baby during your pregnancy?

O None O A little O A good amount O An excellent amount

39. How would you rate the amount of social support you received during your pregnancy from your other family members and your friends?

O None O A little O A good amount O An excellent amount

Study ID____________________________

IV. REPRODUCTIVE HISTORY

40. How old were you when you had your 1st period? [ ][ ] **years**

a. Does your menstrual period come each month? ○ Yes ○ No

b. Does your menstrual period come around the same time (+/- 7 d of LMP)? ○ Yes ○ No

c. What is your average cycle length in days, that is how many days are there from one period to the next? [ ][ ][ ] **days**

d. How long does each period's bleeding last? [ ][ ][ ] **days**

41. Do you have pelvic or abdominal pain during your menstrual period?
○ No ○ Occasionally ○ Almost all the time

42a. If "Occasionally" or "Almost all the time": Do you rate your menstrual pain as:
○ Mild ○ Moderate ○ Severe (could not go to school or work)

42. Prior to this pregnancy, what kind of birth control were you using? (check all that apply)

- ○ **None**
- ○ **Abstinence during fertile days (eg. Natural family planning)**
- ○ **Birth Control Pills**
- ○ **Cervical Cap**
- ○ **Condoms**
- ○ **Creams**
- ○ **Hormone Shots**
- ○ **IUD**
- ○ **Patch**
- ○ **Withdrawel**
- ○ **Other**

If "Other", Specify: [ ]

5884467114

Study ID________________________

43. How many times have you been pregnant (including miscarriage, abortion, stillbirth) ? [ ][ ] X's

What were the results of those pregnancies? (NOT including the index baby)

	1st Pregnancy	2nd Pregnancy	3rd Pregnancy
a. On what date did the pregnancy end?	M [ ][ ] / D [ ][ ] / Y [ ][ ]	M [ ][ ] / D [ ][ ] / Y [ ][ ]	M [ ][ ] / D [ ][ ] / Y [ ][ ]
b. How many weeks did the pregnancy last?	[ ][ ]	[ ][ ]	[ ][ ]
c. How did the pregnancy end?	O Live birth<br>O Still birth<br>O Miscarriage<br>O Abortion<br>O Ectopic pregnancy<br>O Moles	O Live birth<br>O Still birth<br>O Miscarriage<br>O Abortion<br>O Ectopic pregnancy<br>O Moles	O Live birth<br>O Still birth<br>O Miscarriage<br>O Abortion<br>O Ectopic pregnancy<br>O Moles
d. Pregnancy compli-cations?	O None<br>O Mild Preeclampsia<br>O Severe Preeclampsia<br>O Eclampsia<br>O Abruptio placentae<br>O Placenta previa<br>O Incompetent cervix<br>O Gestational diabetes<br>O Intrauterine infection<br>O Others [ ]	O None<br>O Mild Preeclampsia<br>O Severe Preeclampsia<br>O Eclampsia<br>O Abruptio placentae<br>O Placenta previa<br>O Incompetent cervix<br>O Gestational diabetes<br>O Intrauterine infection<br>O Others [ ]	O None<br>O Mild Preeclampsia<br>O Severe Preeclampsia<br>O Eclampsia<br>O Abruptio placentae<br>O Placenta previa<br>O Incompetent cervix<br>O Gestational diabetes<br>O Intrauterine infection<br>O Others [ ]

If this was a live birth, complete the following questions

	1st Pregnancy	2nd Pregnancy	3rd Pregnancy
E. Sex of the baby	O Male O Female	O Male O Female	O Male O Female
f. Type of Delivery	O Vaginal O C-section	O Vaginal O C-section	O Vaginal O C-section
g. Birthweight of the baby	[ ][ ] lb [ ][ ] oz	[ ][ ] lb [ ][ ] oz	[ ][ ] lb [ ][ ] oz
h. Any Birth Defects	[ ]	[ ]	[ ]

43. Continued I

	4th Pregnancy	5th Pregnancy	6th Pregnancy
a. On what date did the pregnancy end?	M ☐☐ / D ☐☐ / Y ☐☐	M ☐☐ / D ☐☐ / Y ☐☐	M ☐☐ / D ☐☐ / Y ☐☐
b. How many weeks did the pregnancy last?	☐☐	☐☐	☐☐
c. How did the pregnancy end?	O Live birth<br>O Still birth<br>O Miscarriage<br>O Abortion<br>O Ectopic pregnancy<br>O Moles	O Live birth<br>O Still birth<br>O Miscarriage<br>O Abortion<br>O Ectopic pregnancy<br>O Moles	O Live birth<br>O Still birth<br>O Miscarriage<br>O Abortion<br>O Ectopic pregnancy<br>O Moles
d. Pregnancy compli-cations?	O None<br>O Mild Preeclampsia<br>O Severe Preeclampsia<br>O Eclampsia<br>O Abruptio placentae<br>O Placenta previa<br>O Incompetent cervix<br>O Gestational diabetes<br>O Intrauterine infection<br>O Others ☐	O None<br>O Mild Preeclampsia<br>O Severe Preeclampsia<br>O Eclampsia<br>O Abruptio placentae<br>O Placenta previa<br>O Incompetent cervix<br>O Gestational diabetes<br>O Intrauterine infection<br>O Others ☐	O None<br>O Mild Preeclampsia<br>O Severe Preeclampsia<br>O Eclampsia<br>O Abruptio placentae<br>O Placenta previa<br>O Incompetent cervix<br>O Gestational diabetes<br>O Intrauterine infection<br>O Others ☐

If this was a live birth, complete the following questions

E. Sex of the baby	O Male O Female	O Male O Female	O Male O Female
f. Type of Delivery	O Vaginal O C-section	O Vaginal O C-section	O Vaginal O C-section
g. Birthweight of the baby	☐☐ lb ☐☐ oz	☐☐ lb ☐☐ oz	☐☐ lb ☐☐ oz
h. Any Birth Defects	☐	☐	☐

Study ID___________________________

43. Continued II

	7th Pregnancy	8th Pregnancy	9th Pregnancy
**a. On what date did the pregnancy end?**	M / D / Y	M / D / Y	M / D / Y
**b. How many weeks did the pregnancy last?**			
**c. How did the pregnancy end?**	O Live birth<br>O Still birth<br>O Miscarriage<br>O Abortion<br>O Ectopic pregnancy<br>O Moles	O Live birth<br>O Still birth<br>O Miscarriage<br>O Abortion<br>O Ectopic pregnancy<br>O Moles	O Live birth<br>O Still birth<br>O Miscarriage<br>O Abortion<br>O Ectopic pregnancy<br>O Moles
**d. Pregnancy compli-cations?**	O None<br>O Mild Preeclampsia<br>O Severe Preeclampsia<br>O Eclampsia<br>O Abruptio placentae<br>O Placenta previa<br>O Incompetent cervix<br>O Gestational diabetes<br>O Intrauterine infection<br>O Others	O None<br>O Mild Preeclampsia<br>O Severe Preeclampsia<br>O Eclampsia<br>O Abruptio placentae<br>O Placenta previa<br>O Incompetent cervix<br>O Gestational diabetes<br>O Intrauterine infection<br>O Others	O None<br>O Mild Preeclampsia<br>O Severe Preeclampsia<br>O Eclampsia<br>O Abruptio placentae<br>O Placenta previa<br>O Incompetent cervix<br>O Gestational diabetes<br>O Intrauterine infection<br>O Others

If this was a live birth, complete the following questions

	7th Pregnancy	8th Pregnancy	9th Pregnancy
**E. Sex of the baby**	O Male O Female	O Male O Female	O Male O Female
**f. Type of Delivery**	O Vaginal O C-section	O Vaginal O C-section	O Vaginal O C-section
**g. Birthweight of the baby**	lb oz	lb oz	lb oz
**h. Any Birth Defects**			

Study ID______________________________

43. Continued III

	10th Pregnancy	11th Pregnancy	12th Pregnancy
a. On what date did the pregnancy end?	M / D / Y	M / D / Y	M / D / Y
b. How many weeks did the pregnancy last?			
c. How did the pregnancy end?	O Live birth<br>O Still birth<br>O Miscarriage<br>O Abortion<br>O Ectopic pregnancy<br>O Moles	O Live birth<br>O Still birth<br>O Miscarriage<br>O Abortion<br>O Ectopic pregnancy<br>O Moles	O Live birth<br>O Still birth<br>O Miscarriage<br>O Abortion<br>O Ectopic pregnancy<br>O Moles
d. Pregnancy compli-cations?	O None<br>O Mild Preeclampsia<br>O Severe Preeclampsia<br>O Eclampsia<br>O Abruptio placentae<br>O Placenta previa<br>O Incompetent cervix<br>O Gestational diabetes<br>O Intrauterine infection<br>O Others	O None<br>O Mild Preeclampsia<br>O Severe Preeclampsia<br>O Eclampsia<br>O Abruptio placentae<br>O Placenta previa<br>O Incompetent cervix<br>O Gestational diabetes<br>O Intrauterine infection<br>O Others	O None<br>O Mild Preeclampsia<br>O Severe Preeclampsia<br>O Eclampsia<br>O Abruptio placentae<br>O Placenta previa<br>O Incompetent cervix<br>O Gestational diabetes<br>O Intrauterine infection<br>O Others

If this was a live birth, complete the following questions

E. Sex of the baby	O Male O Female	O Male O Female	O Male O Female
f. Type of Delivery	O Vaginal O C-section	O Vaginal O C-section	O Vaginal O C-section
g. Birthweight of the baby	lb oz	lb oz	lb oz
h. Any Birth Defects			

Study ID______________________________

44. Did you have sexual intercourse during this pregnancy, in the:

a1. 1st trimester? ○ Yes ○ No

if yes: a2. use condom? ○ Yes ○ No

a3. How often did you have intercourse then? ☐☐ **times/month**

b1. 2nd trimester? ○ Yes ○ No

if yes: b2. use condom? ○ Yes ○ No

b3. How often did you have intercourse then? ☐☐ **times/month**

c1. 3rd trimester? ○ Yes ○ No

if yes: c2. use condom? ○ Yes ○ No

c3. How often did you have intercourse then? ☐☐ **times/month**

45. Did you have more than one sexual partner during this pregnancy? ○ Yes ○ No

46. FOR DATA ENTRY ONLY

a. Gravidity (total # of pregnancies INCLUDING index case) ☐☐

b. Parity (# of live births NOT INCLUDING index case) ☐☐

c. Number of prior perterm births (<37wks) ☐☐

d. Number of prior LBW births (<2500g) ☐☐

e. Number of prior stillbirths ☐☐

f. Number of spontaneous abortions ☐☐

g. Number of induced abortions ☐☐

Study ID____________________________

V. DAILY PHYSICAL ACTIVITY BEFORE AND DURING THE INDEX PREGNANCY

	3 mo. pre-pregnancy	1st trimester	2nd trimester	3rd trimester
**47a. Working**	O Yes O No	O Yes O No	O Yes O No	O Yes O No

If NO, to ALL of question 47a, skip to 48 (next page)

	3 mo. pre-pregnancy	1st trimester	2nd trimester	3rd trimester
**47b. Industry**	[ ]	[ ]	[ ]	[ ]
**47c. Job Title**	[ ]	[ ]	[ ]	[ ]
**47d. Duties**	[ ]	[ ]	[ ]	[ ]
**47e. Your work schedule**	O Not working<br>O Regular day shift<br>O Regular evening shift<br>O Regular night shift<br>O Irregular shifts	O Not working<br>O Regular day shift<br>O Regular evening shift<br>O Regular night shift<br>O Irregular shifts	O Not working<br>O Regular day shift<br>O Regular evening shift<br>O Regular night shift<br>O Irregular shifts	O Not working<br>O Regular day shift<br>O Regular evening shift<br>O Regular night shift<br>O Irregular shifts
**47f. How many hours did you work each week?**	[ ][ ]	[ ][ ]	[ ][ ]	[ ][ ]
**47g. How long did it take to get to work? (one-way) in MINUTES**	[ ][ ][ ]	[ ][ ][ ]	[ ][ ][ ]	[ ][ ][ ]
**47h. How physically demanding is your job?**	O Slight<br>O Moderate<br>O Very Much	O Slight<br>O Moderate<br>O Very Much	O Slight<br>O Moderate<br>O Very Much	O Slight<br>O Moderate<br>O Very Much
**47i. How much job-related mental stress did you experience?**	O Slight<br>O Moderate<br>O Very Much	O Slight<br>O Moderate<br>O Very Much	O Slight<br>O Moderate<br>O Very Much	O Slight<br>O Moderate<br>O Very Much

VI. HOME ENVIRONMENT

48. Did you live outside the U.S. during this(index) pregnancy? O Yes O No

IF YES **a. What country(s) did you live in?** [ ]

Country Code: [ ][ ][ ]

b. For how long did you live outside the U.S.? [ ][ ] **weeks**

c. (Calculate, do not ask) Most of pregnancy was: O Inside U.S. O Outside U.S.

49. IF LIVED MOST OF PREGNANCY IN U.S., ASK:

a. What was the zip code of the place you lived longest? [ ][ ][ ][ ][ ]

b. If don't know zip code: What town was it? [ ]

50. How long have you lived in your current home?

[ ][ ] **years** [ ][ ] **months** [ ][ ] **days**

51. Did you live in a shelter for any part of this pregnancy? O Yes O No

If YES, how long? [ ][ ] **months** [ ][ ] **weeks** [ ][ ] **days**

52. All questions below refer to the home mother lived the longest

52a. # of bedrooms [ ][ ]

52b. # of bathrooms [ ][ ]

52c. # of people who permanently live in your home [ ][ ][ ]

52d. what type of fuel do you use for heating your home?

O Oil O Electricity O Gas O Other **, specify** [ ]

52e. What type of stove did you use for cooking?

O Gas O Electric O Other **, specify** [ ]

52f. Do you have any wall to wall carpet in your home? O Yes O No

if yes, specify location:

O Living room O Family room
O Dinning room O Kitchen
O Bedroom O Basement
O Bathroom O Hallways
O Other

If Other, specify [ ]

Do/did you have any pets at the place you lived at the longest? O Yes O No

52g. Cat O Yes O No
52h. Dog O Yes O No
52i. Fish O Yes O No
52j. Birds O Yes O No
52k. Reptiles O Yes O No
52l. Rabbit O Yes O No
52m. Guinea pig O Yes O No
52n. Other O Yes O No
if Other, specify [ ]

52o. Does the house you lived in the longest have any cockroaches? O Yes O No

52p. Does the house you lived in the longest have any mice/rats? O Yes O No

53q. Does the house you lived in the longest have any visible mold, mildew, water damage, leakage or seepage? O Yes O No

52r. Was the place you lived in the longest a farming environment? O Yes O No

VII. CIGARETTE SMOKING

53. Have you ever smoked cigarettes, cigars, or pipe tobacco, or used chewing tobacco or snuff? (Even once counts) O Yes O No

If NO, skip to question 75 (second-hand smoke) on page 22

54. Have you ever smoked or used tobacco regularly for at least a month? O Yes O No

If NO, skip to question 74 (smoking during pregnancy) on page 21

55. How old were you when you began to smoke or use tobacco regularly? [|] years

56. Altogether, over your entire life, how long have you smoked or used tobacco regularly?

[|] years [|] months

57. When you used tobacco regularly, did you use cigarettes, cigars, pipes, chewing tobacco, or snuff?

a. Cigarettes O Yes O No
if yes: When you smoked cigarettes, on average how many cigarettes would you smoke per day? [|] cigarettes

b. Cigars O Yes O No
if yes: When you smoked cigars, on average how many cigars would you smoke per day? [|] cigars

c. **Pipes** O Yes O No

if yes: When you smoked pipes, on average how many pipes would you smoke per day? [|] **pipes**

d. **Chewing tobacco** O Yes O No

if yes: When you chewed tobaccos, on average how much would you use per day? [|] **chaws**

e. **Snuff** O Yes O No

if yes: When you used snuff, on average how much would you use per day? [|] **dips**

58. I would now like to ask you some questions about your (smoking/tobacco use) during the time in your life when you were using tobacco the most heavily. How old were you at that time? (IF OVER A PERIOD OF TIME RECORD AGE AT WHICH BEGAN USING HEAVILY)

[|] **years**

59. During the time when you were(smoking / using tobacco) most heavily, on average, how many _______________ would you have per day?

a. Cigarettes: [|] **b. Cigars:** [|] **c. Pipes:** [|]

d. Dips: [|] **e. Chaws:** [|]

60. During this time when you (smoked / used tobacco) most heavily, how soon after you awoke did you (smoke / used tobacco)?

[|] **hours** [|] **minutes**

61. During this time when you (smoked / used tobacco) most heavily, how often would you check to make sure that you had (cigarettes/cigars/tobacco) around to (smoke / use)?

Would you say... O Often O Sometimes O Rarely O Never

62. During this time when you (smoked / used tobacco) most heavily, if you didn't (smoke / use tobacco) for a period of time, how strong would your craving get for another (cigarette / cigar / pipe / dip / chaw)? O Very Strong O Strong O Moderate O Hardly any

63. During this time when you (smoked / used tobacco) most heavily, how difficult was it for you to not (smoke / use) it in places where it was forbidden? Would you say...

O Very difficult O Somewhat difficult O A little difficult O Not difficult at all

64. During this time when you (smoked / used tobacco) most heavily, would you (smoke / use tobacco) when you were so ill that you were in bed most of the day? O Yes O No

65. During this time when you (smoked / used tobacco) most heavily, would you (smoke / use tobacco) more during the morning than during the rest of the day? O Yes O No

66. During this time when you (smoked / used tobacco) most heavily, which (cigarette / cigar / pipe / dip / chaw) of the day would be the most satisfying? Was it the first?

O First O Other O Not sure

67. IF SMOKED IN HEAVIEST USE PERIOD: During the time when you smoked most heavily, how often did you inhale? Would you say: O Always O Sometimes O Never

68a. IF ALWAYS OR SOMETIMES: How often did you inhale deep into your lungs? Would you say: O Always O Sometimes O Never

68. Have you ever seriously attempted to stop (smoking / using tobacco)? O Yes O No

If NO, skip to question 74 (smoking during pregnancy) on page 21

69. How many times in you life have you seriously tried to stop (smoking / using tobacco?

		**times**

70. How depressed did you get when you tried to quit (smoking / using tobacco)?

O Very O Somewhat O A little O Hardly at all

72. How nervous, jittery, or irritable did you get when you tried to quit (smoking / using tobacco)?

O Very O Somewhat O A little O Hardly at all

71. Have you ever gone to a professional to help you stop (smoking / using tobacco)? O Yes O No

IF YES: Whom did you see? (circle all that apply)

O a. Regular doctor
O b. Mental health professional
O c. Stop smoking clinic / workshop
O d. Hypnotist
O e. Other

if other, specify [ ]

73. Have you ever used nicotine gum or patches to help you stop (smoking / using tobacco)?
○ Yes ○ No

74. I'd like to ask you some questions about your (smoking / tobacco use) before you found out you were pregnant.

a. In the six months before you found out you were pregnant, did you (smoke / use tobacco)?
○ Yes ○ No

If yes, On average, how many ______________ did you have per day?

a. Cigarettes: ☐☐ b. Cigars: ☐☐ c. Pipes: ☐☐

d. Dips: ☐☐ e. Chaws: ☐☐

b. In the first three months of your pregnancy, did you (smoke / use tobacco)? ○ Yes ○ No

If yes, On average, how many ______________ did you have per day?

a. Cigarettes: ☐☐ b. Cigars: ☐☐ c. Pipes: ☐☐

d. Dips: ☐☐ e. Chaws: ☐☐

c. In the middle three months of your pregnancy, did you (smoke / use tobacco)? ○ Yes ○ No

If yes, On average, how many ______________ did you have per day?

a. Cigarettes: ☐☐ b. Cigars: ☐☐ c. Pipes: ☐☐

d. Dips: ☐☐ e. Chaws: ☐☐

d. In the last three months of your pregnancy, did you (smoke / use tobacco)? ○ Yes ○ No

If yes, On average, how many ______________ did you have per day?

a. Cigarettes: ☐☐ b. Cigars: ☐☐ c. Pipes: ☐☐

d. Dips: ☐☐ e. Chaws: ☐☐

75. How many people who live in your home smoke cigarettes (NOT counting yourself)? [|]

76. How many of them smoke inside the home? [|]

77. Total numbers of cigarettes smoked inside your home per day (NOT including amount subject smoked) [|] cigs/day

VIII. ALCOHOL AND DRUG USE

I'd like to ask you some questions about alcohol and drinking

78. In the six months before you found out you were pregnant, how often did you drink?

O Never O Occasionally (special occasions / holidays) O Regularly

If Regularly:

a. How many drinks did you have in a typical week? [|]

b. What type of drinks were they? (NUMBER OF EACH)

[|] beers or wine coolers [|] glasses of wine

[|] shots of liquor [|] mixed drinks

IF MIXED DRINKS: How much alcohol was in each drink? [ ]

79. In the first three months of your pregnancy, how often did you drink?

O Never O Occasionally (special occasions / holidays) O Regularly

If Regularly:

a. How many drinks did you have in a typical week? [|]

b. What type of drinks were they? (NUMBER OF EACH)

[|] beers or wine coolers [|] glasses of wine

[|] shots of liquor [|] mixed drinks

IF MIXED DRINKS: How much alcohol was in each drink? [ ]

80. In the middle three months of your pregnancy, how often did you drink?

O Never O Occasionally (special occasions / holidays) O Regularly

If Regularly:

a. How many drinks did you have in a typical week? [ ][ ]

b. What type of drinks were they? (NUMBER OF EACH)

[ ][ ] **beers or wine coolers** [ ][ ] **glasses of wine**

[ ][ ] **shots of liquor** [ ][ ] **mixed drinks**

IF MIXED DRINKS: How much alcohol was in each drink? [ ]

81. In the last three months of your pregnancy, how often did you drink?

O Never O Occasionally (special occasions / holidays) O Regularly

If Regularly:

a. How many drinks did you have in a typical week? [ ][ ]

b. What type of drinks were they? (NUMBER OF EACH)

[ ][ ] **beers or wine coolers** [ ][ ] **glasses of wine**

[ ][ ] **shots of liquor** [ ][ ] **mixed drinks**

IF MIXED DRINKS: How much alcohol was in each drink? [ ]

82. Now I'd like to ask you some questions about drug use. Have you ever used..(read each one)

a. Marijuana O Yes O No

b. Heroin O Yes O No **If yes, have you ever been on methadone treatment?** O Yes O No

c. Cocaine O Yes O No

d. Crack O Yes O No

e. Speed/Amphetamines O Yes O No

f. Paint/Glue inhalant O Yes O No

g. PCP O Yes O No

h. Barbituates O Yes O No

i. Benzo's/Valium O Yes O No

j. Ecstasy O Yes O No

k. LSD/Hallucinogens O Yes O No

l. Oxycodone O Yes O No

m. Other O Yes O No **specify:** [ ]

4736467119

Study ID______________________________

83. If used any drug WITHIN 6 months pre-pregnancy and during CURRENT (index) pregnancy, fill out chart BELOW:
CODE: 1=occasional; 2=regular ; If regular, write in amount X/wk

Drugs	6 mo pre-pregnancy	1st trimester	2nd trimester	3rd trimester
Marijuana	O 1 O 2 ☐☐ X/WK	O 1 O 2 ☐☐ X/WK	O 1 O 2 ☐☐ X/WK	O 1 O 2 ☐☐ X/WK
Heroin	O 1 O 2 ☐☐ X/WK	O 1 O 2 ☐☐ X/WK	O 1 O 2 ☐☐ X/WK	O 1 O 2 ☐☐ X/WK
Methadone	O 1 O 2 ☐☐ X/WK	O 1 O 2 ☐☐ X/WK	O 1 O 2 ☐☐ X/WK	O 1 O 2 ☐☐ X/WK
Cocaine	O 1 O 2 ☐☐ X/WK	O 1 O 2 ☐☐ X/WK	O 1 O 2 ☐☐ X/WK	O 1 O 2 ☐☐ X/WK
Crack	O 1 O 2 ☐☐ X/WK	O 1 O 2 ☐☐ X/WK	O 1 O 2 ☐☐ X/WK	O 1 O 2 ☐☐ X/WK
Speed/Am phetamine	O 1 O 2 ☐☐ X/WK	O 1 O 2 ☐☐ X/WK	O 1 O 2 ☐☐ X/WK	O 1 O 2 ☐☐ X/WK
Paint/Glue	O 1 O 2 ☐☐ X/WK	O 1 O 2 ☐☐ X/WK	O 1 O 2 ☐☐ X/WK	O 1 O 2 ☐☐ X/WK
PCP	O 1 O 2 ☐☐ X/WK	O 1 O 2 ☐☐ X/WK	O 1 O 2 ☐☐ X/WK	O 1 O 2 ☐☐ X/WK

**Barbituates**	O 1 O 2 [ ][ ] **X/WK**	O 1 O 2 [ ][ ] **X/WK**	O 1 O 2 [ ][ ] **X/WK**	O 1 O 2 [ ][ ] **X/WK**
**Benzo's/ Valium**	O 1 O 2 [ ][ ] **X/WK**	O 1 O 2 [ ][ ] **X/WK**	O 1 O 2 [ ][ ] **X/WK**	O 1 O 2 [ ][ ] **X/WK**
**Ecstasy**	O 1 O 2 [ ][ ] **X/WK**	O 1 O 2 [ ][ ] **X/WK**	O 1 O 2 [ ][ ] **X/WK**	O 1 O 2 [ ][ ] **X/WK**
**LSD/Hallu cinogen**	O 1 O 2 [ ][ ] **X/WK**	O 1 O 2 [ ][ ] **X/WK**	O 1 O 2 [ ][ ] **X/WK**	O 1 O 2 [ ][ ] **X/WK**
**Other**	O 1 O 2 [ ][ ] **X/WK**	O 1 O 2 [ ][ ] **X/WK**	O 1 O 2 [ ][ ] **X/WK**	O 1 O 2 [ ][ ] **X/WK**

If other, please specify drug: [ ]

IX. GENERAL INFORMATION

84. How much did you weigh when you were born?

[ ][ ] **lbs** [ ][ ] **oz** **OR** [ ][ ][ ][ ] **grams**

85. Were you born prematurely? O Yes O No

85a. If yes, at what gestation? [ ][ ] **weeks**

86. What is the highest grade of school you have completed?

- O No school / Elementary school
- O Some secondary school (9th grade and above)
- O High school graduate or GED
- O Some college
- O College degree and above

87. Which one of these groups best describes your racial background?

O Black / African American O White O Hispanic
O Asian O Haitian O Cape Verdian
O Pacific Islander O Other O Unknown

88. Where were you born? O U.S. O Foreign country(specify) O Unknown

Other Country: [ ] **Country Code :** [| |]

88a. If FOREIGN BORN: How long have you lived in the U.S.?

[|] **years** [|] **months** [|] **days**

89. Where was your mother born? O U.S. O Foreign country(specify) O Unknown

Other Country: [ ] **Country Code :** [| |]

90. Where was your father born? O U.S. O Foreign country(specify) O Unknown

Other Country: [ ] **Country Code :** [| |]

91. What is your native language?

O English O Spanish O Haitian Creole O French O Portuguese O Other

91a. If not English: How would you rate your ability to speak English?

O Very well O Well O Not very well O Not at all

92. Will you answer some questions about your child's biological father? O Yes O No

If NO, skip to question 96 on next page

93. What is the highest grade of school the baby's father has completed?

O No school / Elementary school O Some secondary school (9th grade and above)
O High school graduate or GED O Some college
O College degree and above O Unknown

94. Which one of these groups best describes the racial background of the baby's father?

O Black / African American O White O Hispanic
O Asian O Haitian O Cape Verdian
O Pacific Islander O Other O Unknown

95. Where was the baby's father born? O U.S. O Foreign country(specify) O Unknown

Other Country: [ ] **Country Code :** [| |]

Study ID____________________________

96. What is your present marital status?

O Married O Widowed O Divorced O Separated O Single

97. What was your total household income last year, before taxes? (Includes public assistance)

O <$5,000 O $5000 - 9,999 O $10,000 - 14,999 O $15,000 - 19,999

O $20,000 - 24,999 O $25,000 - 29,999 O $30,000 - 34,999 O $35,000 - 39,999

O $40,000 - 49,999 O $50,000 - 59,999 O $60,000 and over O Don't know

97a. Please ask if mother does not know annual income only:

What is your weekly income? $ [ ][ ][ ][ ]

98. Are you getting:

a. WIC? O Yes O No **b. Food Stamps?** O Yes O No

c. AFDC? (Aid to families with dependent children) O Yes O No

d. Housing assistance? O Yes O No **e. Fuel assistance?** O Yes O No

f. Any other public assistance? O Yes O No **if other, specify** [ ]

X. DIETARY HISTORY

99. Did you take prenatal vitamins prescribed by your doctor? O Yes O No

(prepregnancy -- 6 month prior to conception)

a. Prepregnancy O No O <1x/wk O 1-2x/wk O 3-5x/wk O Almost daily

b. 1st trimester O No O <1x/wk O 1-2x/wk O 3-5x/wk O Almost daily

c. 2nd trimester O No O <1x/wk O 1-2x/wk O 3-5x/wk O Almost daily

d. 3rd trimester O No O <1x/wk O 1-2x/wk O 3-5x/wk O Almost daily

100. Did you take iron? O Yes O No

(prepregnancy -- 6 month prior to conception)

a. Prepregnancy O No O <1x/wk O 1-2x/wk O 3-5x/wk O Almost daily

b. 1st trimester O No O <1x/wk O 1-2x/wk O 3-5x/wk O Almost daily

c. 2nd trimester O No O <1x/wk O 1-2x/wk O 3-5x/wk O Almost daily

d. 3rd trimester O No O <1x/wk O 1-2x/wk O 3-5x/wk O Almost daily

101. Did you take any over the counter vitamins? O Yes O No

(prepregnancy -- 6 month prior to conception)

a. Prepregnancy O No O <1x/wk O 1-2x/wk O 3-5x/wk O Almost daily

b. 1st trimester O No O <1x/wk O 1-2x/wk O 3-5x/wk O Almost daily

c. 2nd trimester O No O <1x/wk O 1-2x/wk O 3-5x/wk O Almost daily

d. 3rd trimester O No O <1x/wk O 1-2x/wk O 3-5x/wk O Almost daily

Name of Vitamin: [ ]

Study ID____________________________

102. Did you take any herbal supplements? O Yes O No
(prepregnancy -- 6 month prior to conception)

a. Prepregnancy O No O <1x/wk O 1-2x/wk O 3-5x/wk O Almost daily

b. 1st trimester O No O <1x/wk O 1-2x/wk O 3-5x/wk O Almost daily

c. 2nd trimester O No O <1x/wk O 1-2x/wk O 3-5x/wk O Almost daily

d. 3rd trimester O No O <1x/wk O 1-2x/wk O 3-5x/wk O Almost daily

Name of herbal supplement: [ ]

Name of herbal supplement: [ ]

Name of herbal supplement: [ ]

103. During this pregnancy, on average, how often do you eat or drink following foods per week?

a. Green vegetables O None O <1 days O 1-2 days O 3-5 days O 6-7 days O don't know

b. Orange Veggies (Carrots ,Squash, etc)
O None O <1 days O 1-2 days O 3-5 days O 6-7 days O don't know

c. Fruits O None O <1 days O 1-2 days O 3-5 days O 6-7 days O don't know

d. Meats O None O <1 days O 1-2 days O 3-5 days O 6-7 days O don't know

e. Shellfish O None O <1 days O 1-2 days O 3-5 days O 6-7 days O don't know

f. Fish O None O <1 days O 1-2 days O 3-5 days O 6-7 days O don't know

g. Eggs O None O <1 days O 1-2 days O 3-5 days O 6-7 days O don't know

h. Cow's Milk / Dairy Products / Cheese
O None O <1 days O 1-2 days O 3-5 days O 6-7 days O don't know

i. Beans O None O <1 days O 1-2 days O 3-5 days O 6-7 days O don't know

j. Rice O None O <1 days O 1-2 days O 3-5 days O 6-7 days O don't know

k. Wheat (Pasta, Bread, Cereal)
O None O <1 days O 1-2 days O 3-5 days O 6-7 days O don't know

l. Soy / Tofu O None O <1 days O 1-2 days O 3-5 days O 6-7 days O don't know

m. Seeds (Sesame, Sunflower, Pumpkin)
O None O <1 days O 1-2 days O 3-5 days O 6-7 days O don't know

n. Calcium Fortified Juice O None O <1 days O 1-2 days O 3-5 days O 6-7 days O don't know

o. Peanut O None O <1 days O 1-2 days O 3-5 days O 6-7 days O don't know

p. Tree nuts O None O <1 days O 1-2 days O 3-5 days O 6-7 days O don't know

104. Did you drink coffee before or during the index pregnancy? O Yes O No

If yes, was it regular or decaffeinated? O Regular O Decaffeinated O Both

**How much did you drink?**	**Regular**		**Decaf**	
**1. Prepregnancy**	☐☐☐	**cups/wk**	☐☐☐	**cups/wk**
**2. 1st trimester**	☐☐☐	**cups/wk**	☐☐☐	**cups/wk**
**3. 2nd trimester**	☐☐☐	**cups/wk**	☐☐☐	**cups/wk**
**4. 3rd trimester**	☐☐☐	**cups/wk**	☐☐☐	**cups/wk**

105. Did you drink tea before or during the index pregnancy? O Yes O No

If yes, was it regular tea or herbal tea? O Regular O Herbal O Both

**How much did you drink?**	**Regular**		**Herbal**	
**1. Prepregnancy**	☐☐☐	**cups/wk**	☐☐☐	**cups/wk**
**2. 1st trimester**	☐☐☐	**cups/wk**	☐☐☐	**cups/wk**
**3. 2nd trimester**	☐☐☐	**cups/wk**	☐☐☐	**cups/wk**
**4. 3rd trimester**	☐☐☐	**cups/wk**	☐☐☐	**cups/wk**

106. Did you drink soft drinks during the index pregnancy? O Yes O No

If yes, what kinds? O Caffeinated O Decaf O Both

(If Coke, Pepsi, and/or Mountain Dew) How much (Coke/Pepsi/Mountain Dew)did you drink?

**1. Prepregnancy**	☐☐☐	**cups/wk**
**2. 1st trimester**	☐☐☐	**cups/wk**
**3. 2nd trimester**	☐☐☐	**cups/wk**
**4. 3rd trimester**	☐☐☐	**cups/wk**

107. Do you plan to feed you baby :

O Breast Feed Only O Formula Feed Only O Both Breast Feed and Formula Feed O Don't know yet

XI. MEDICAL HISTORY

108. What medicines did you take during your pregnancy excluding vitamins?

a. Medication name: [ ]

Used in: 1st trimester: O Yes O No O Unsure
2nd trimester: O Yes O No O Unsure
3rd trimester: O Yes O No O Unsure

b. Medication name: [ ]

Used in: 1st trimester: O Yes O No O Unsure
2nd trimester: O Yes O No O Unsure
3rd trimester: O Yes O No O Unsure

c. Medication name: [ ]

Used in: 1st trimester: O Yes O No O Unsure
2nd trimester: O Yes O No O Unsure
3rd trimester: O Yes O No O Unsure

d. Medication name: [ ]

Used in: 1st trimester: O Yes O No O Unsure
2nd trimester: O Yes O No O Unsure
3rd trimester: O Yes O No O Unsure

e. Medication name: [ ]

Used in: 1st trimester: O Yes O No O Unsure
2nd trimester: O Yes O No O Unsure
3rd trimester: O Yes O No O Unsure
